# Intelligent plantar pressure offloading for the prevention of diabetic foot ulcers and amputations

**DOI:** 10.3389/fendo.2023.1166513

**Published:** 2023-07-04

**Authors:** Sarah L. Hemler, Sofia Lydia Ntella, Kenny Jeanmonod, Christian Köchli, Bhawnath Tiwari, Yoan Civet, Yves Perriard, Zoltan Pataky

**Affiliations:** ^1^ Faculty of Medicine, University of Geneva, Geneva, Switzerland; ^2^ Unit of Therapeutic Patient Education, WHO Collaborating Centre, Division of Endocrinology, Diabetology, Nutrition and Therapeutic Patient Education, Geneva University Hospitals, Geneva, Switzerland; ^3^ Integrated Actuators Laboratory (LAI), École polytechnique fédérale de Lausanne (EPFL), Neuchâtel, Switzerland; ^4^ Faculty Diabetes Centre, Faculty of Medicine, University of Geneva, Geneva, Switzerland

**Keywords:** diabetes mellitus, pressure offloading, foot ulcers, amputations, medical device, human factors, smart insole

## Abstract

The high prevalence of lower extremity ulceration and amputation in people with diabetes is strongly linked to difficulties in achieving and maintaining a reduction of high plantar pressures (PPs) which remains an important risk factor. The effectiveness of current offloading footwear is opposed in part by poor patient adherence to these interventions which have an impact on everyday living activities of patients. Moreover, the offloading devices currently available utilize primarily passive techniques, whereas PP distribution is a dynamically changing process with frequent shifts of high PP areas under different areas of the foot. Thus, there is a need for pressure offloading footwear capable of regularly and autonomously adapting to PPs of people with diabetes. The aim of this article is to summarize the concepts of intelligent pressure offloading footwear under development which will regulate PPs in people with diabetes to prevent and treat diabetic foot ulcers. Our team is creating this intelligent footwear with an auto-contouring insole which will continuously read PPs and adapt its shape in the forefoot and heel regions to redistribute high PP areas. The PP-redistribution process is to be performed consistently while the footwear is being worn. To improve adherence, the footwear is designed to resemble a conventional shoe worn by patients in everyday life. Preliminary pressure offloading and user perceptions assessments in people without and with diabetes, respectively, exhibit encouraging results for the future directions of the footwear. Overall, this intelligent footwear is designed to prevent and treat diabetic foot ulcers while enhancing patient usability for the ultimate prevention of lower limb amputations.

## 1 Introduction

There are currently 537 million adults with diabetes mellitus representing 10.5% of the global adult population (1). On average, 19-34% of people with diabetes will develop at least one ulcer in their lifetime (2–4) and 84% of amputations in people with diabetes are due to an ulcer (5). Foot plantar ulcers typically form due to elevated plantar pressures (PPs) as a consequence of peripheral sensorimotor neuropathy (6). Peripheral neuropathy is characterized by the lack of protective pain sensation (the “gift of pain” (7)) and affects up to 50% of individuals with type 2 diabetes (8). Furthermore, ill-fitting footwear has been identified as the root cause of 21-76% of ulcers and/or lower extremity amputations in people with diabetes (9). Effectively implementing pressure offloading interventions is essential for treating and preventing diabetic foot ulcers.

**Figure 1:**
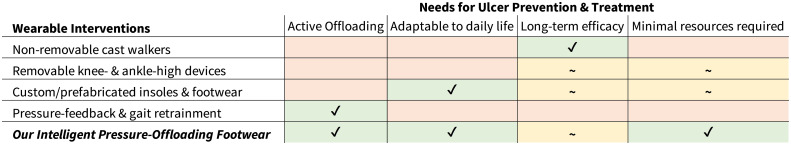
Offloading interventions and the ulcer prevention and treatment needs which each fulfill with proven success (green check marks) or varied/inconsistent success (yellow tilde), or which they do not fulfill (blank orange). Our intelligent footwear plans will meet all needs and will be tested for long-term efficacy.

There are many wearable offloading interventions with varying efficacy and with limitations (**Figure 1**). Non-removable interventions effectively aid ulcer healing because of their forced adherence (10–13). However, these interventions are used in less than 2% of diabetic foot centers and may increase pre-existing challenges such as gait and balance impairments (14–16), low-weight bearing activities (14), restrictions of daily activities, low quality of life (17), and stigmatization (16). Removable interventions include a range of foot enclosures that allow the patient to have autonomy for removal during treatment. In general, removable interventions have been preferred by patients for convenience among other factors (18). Such interventions include knee-high or ankle-high offloading devices and types of footwear and insoles. These interventions vary in effectiveness for reducing peak PP at the ulcer location (10) and thus, aiding in ulcer healing (19). Knee- and ankle-high offloading devices (i.e., cast shoes, half-shoes, and forefoot offloading shoes) have been shown to have higher effectiveness in healing ulcers than conventional or custom-insole footwear (10, 11).

However, knee- and ankle-high offloading devices often limit mobility or have negative rocker outsoles which may induce balance problems (11). Therefore, once the ulcer is healed, lower-height modalities (e.g., custom footwear) which support more natural gait and mobility may be a more practical, long-term solution for ulcer prevention for this population for whom there may already be limited joint mobility (11, 13, 20, 21).

There are a range of therapeutic footwear interventions that have shown varied efficacy in ulcer treatment and prevention. Footwear interventions may include fully customized footwear (i.e., custom insoles in custom-made shoes), semi-customized footwear (i.e., custom insoles in extra-depth shoes), or un-customized footwear (i.e., prefabricated insoles in normal shoes) (22, 23). Custom-insoles are designed to offload high pressure areas for preventing ulcers, and/or to offload known ulcer areas. However, there is varied efficacy in how much pressure reduction occurs in different types of custom insoles (22, 24). Other research has shown a lack of difference in peak PP reduction between custom and prefabricated insoles (23). Overall, there are wide-ranging levels of PP reduction in various types of interventions.

Pressure biofeedback insoles have been used to actively detect and display high pressure areas under the foot. In current systems, users are instructed to adjust their gait to reduce the PPs in the identified regions. These insoles have been shown to be helpful for redistributing pressures, though the method of redistribution involves patient’s active participation which is difficult in the long-term (25–27). In one of these studies, the learning response took 12 weeks of wear (25). Thus, there is a need for a footwear strategy that actively senses and instantly offloads the high plantar regions without cognitive input from the user.

Therapeutic footwear could have high adherence if designed according to the desires of the patient. Offloading intervention adherence is associated with ulcer healing (28) and wearing offloading footwear for the majority of the day has been shown to reduce the risk for foot re-ulceration (29, 30). However, studies have shown that less than 50% of patients wear their therapeutic footwear for more than 60% of daytime hours (31, 32). Furthermore, one study showed that among the most important footwear features for patients with diabetes, style was a priority compared to comfort as a priority in people with other diseases such as rheumatoid arthritis (33). Important factors influencing therapeutic footwear dissatisfaction for people with diabetes and neuropathy are the weight (31, 34) and comfort of the footwear (31), and the perceived opinion of others (35). Thus, improving the design and style of therapeutic footwear to be adapted to the patients’ desires, is mandatory to improve long-term adherence.

Overall, there is a need for user-friendly, offloading footwear that reduces high PP to prevent and treat foot ulcers. The aim of this article is to present a review of the advancements toward this goal made by a multi-centered team from the Geneva University Hospitals (HUG), University of Geneva (UNIGE), and École polytechnique fédérale de Lausanne (EPFL). The team is developing intelligent offloading footwear that is designed to use a pressure feedback loop to automatically sense and redistribute PPs to prevent and treat diabetic foot ulcers (36).

## 2 Intelligent footwear design

### 2.1 Summary of pressure-offloading

**Figure 2:**
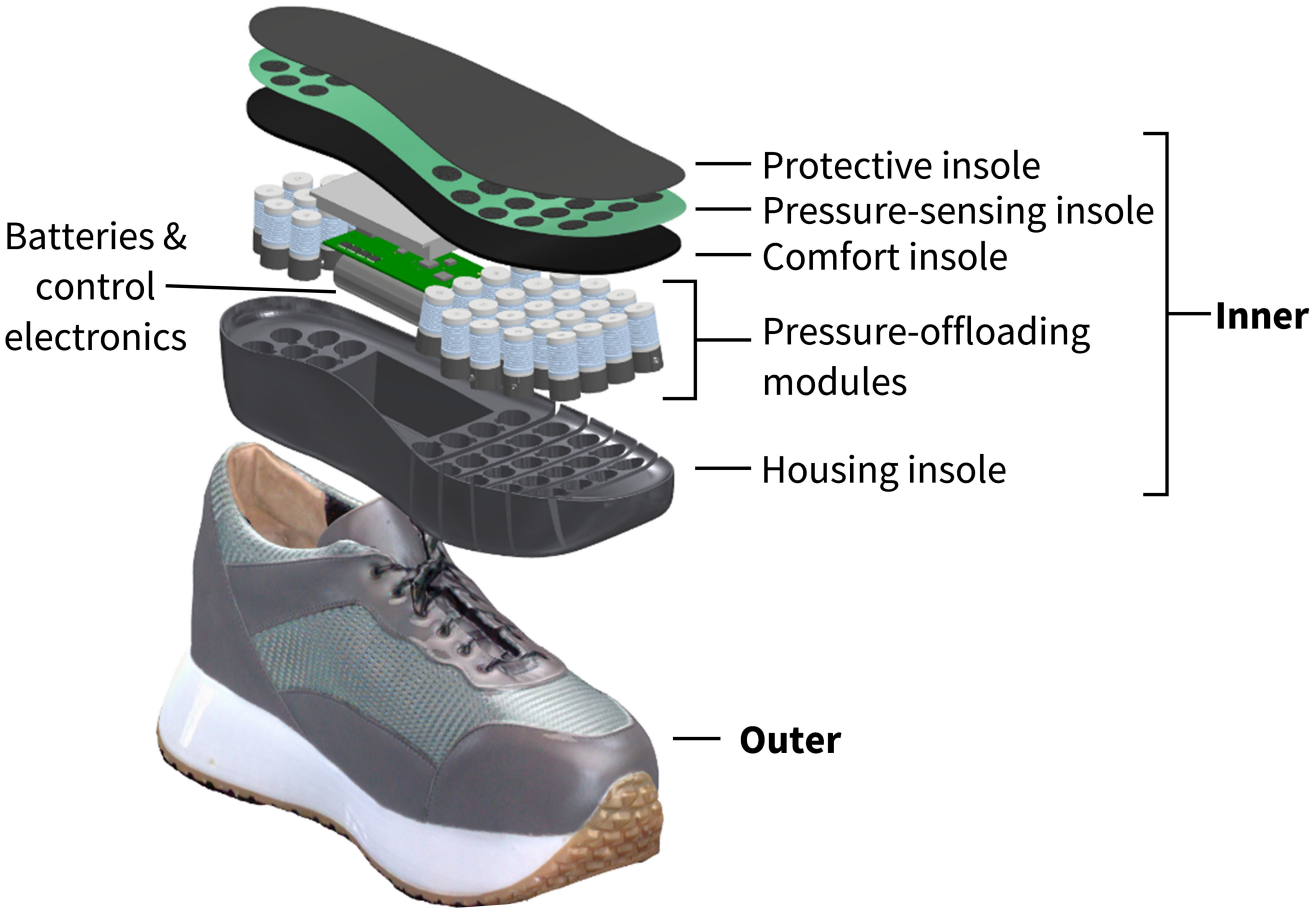
Schematic of the outer and inner (removable insole system) parts of the shoe. The removable insole system includes the housing insole which contains the batteries, computing device, and pressure-offloading modules, and the comfort insole, pressure-sensing insole, and protective insole which rest on top of the housing insole.

**Figure 3:**
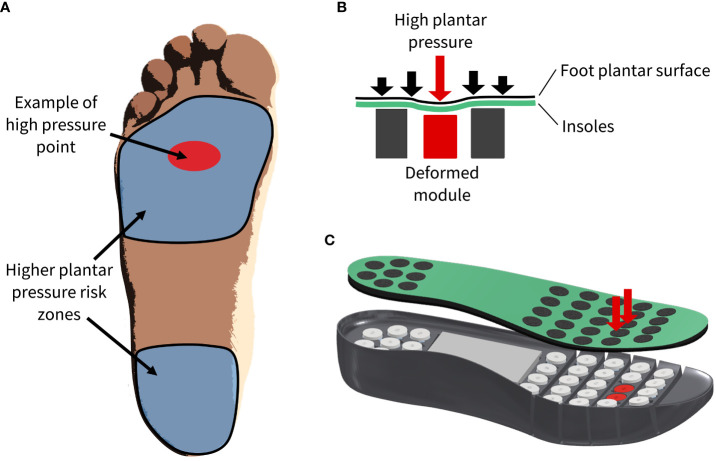
**(A)** High pressure regions of interest and example high pressures on the plantar surface of the foot. **(B)** 2D, **(C)** 3D schematics of high plantar pressures (downward red arrows) which guide the automation and deformation of specific modules (in red).

The intelligent footwear presented in this article consists of outer and inner (removable insole system) parts (**Figure 2**). Within the removable insole system, there is a pressure-sensing system coupled with miniaturized pressure-offloading modules. The system is designed to automatically detect the location of high PPs and correspondingly adjust the contour of the insole according to the user’s individual pressure needs (**Figure 3**).

### 2.2 Intelligent insole system

**Figure 4:**
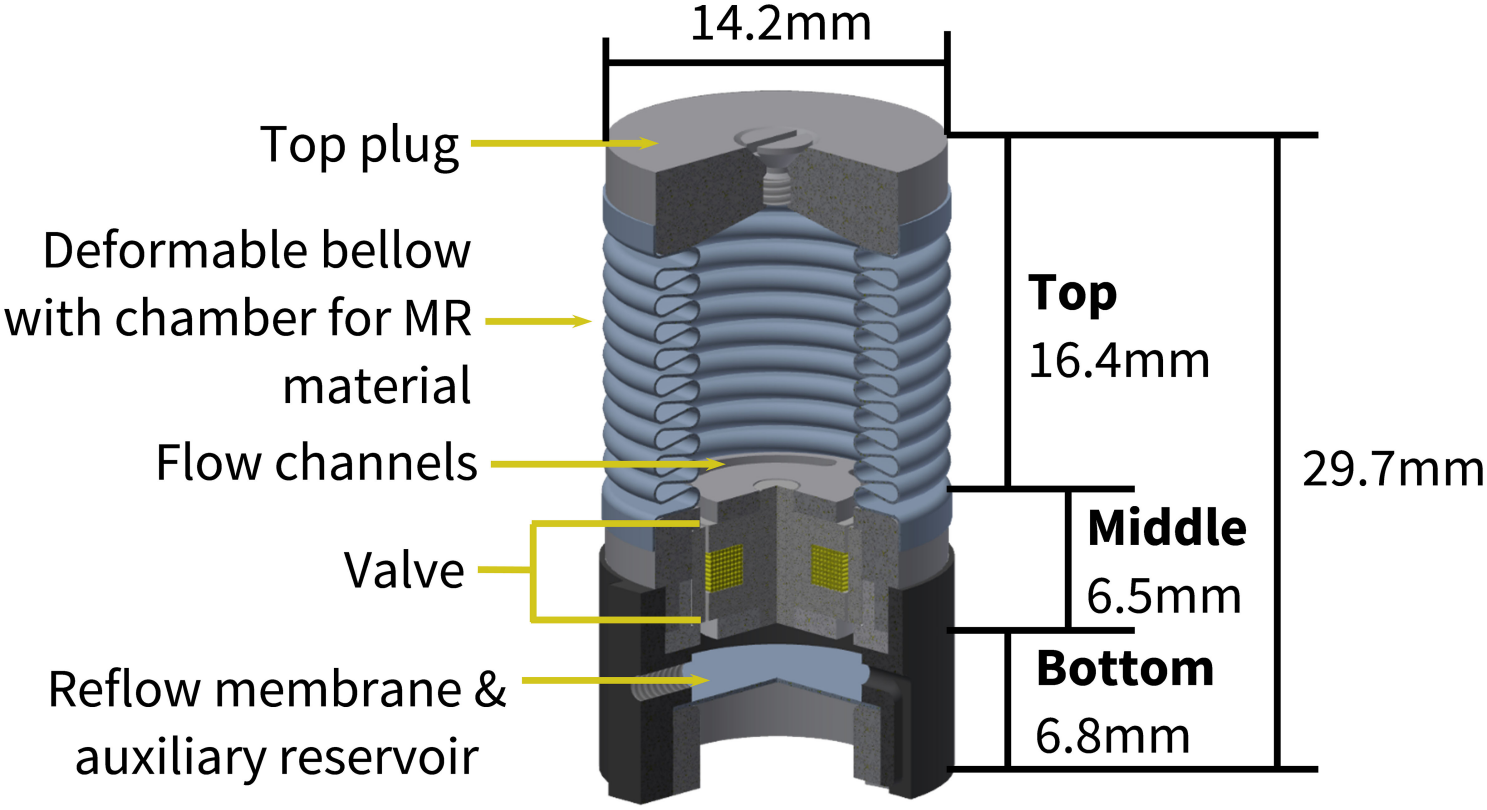
The pressure-offloading module frame and dimensions.

Inside the footwear, there is a removable, intelligent insole system (**Figure 2** - inner), which is made of several components working together to redistribute high PP. The system consists of a housing insole in which the pressure-offloading modules, batteries, and control electronics rest, and above which the comfort insole and pressure-sensing insole sit. The pressure-offloading modules operate independently and are connected to the control electronics via flex PCB through channels on the underside of the housing insole. Each pressure-offloading module consists of three primary parts: 1) Top - deformable bellow filled with magnetorheological (MR) fluid and top plug, 2) Middle – flow channels and valve, and 3) Bottom - deformable reflow membrane and auxiliary reservoir (**Figure 4**) (37). The pressure-sensing insole consists of piezoresistive sensors (dynamic range: 0-800kPa; sampling frequency: 200 Hz) aligned directly over each corresponding pressure-offloading module. The thin-protective layer is the interface between the foot and the removable insole system with a goal of providing a moisture-absorbing and comfortable barrier between the mechanics and the foot.

**Figure 5:**
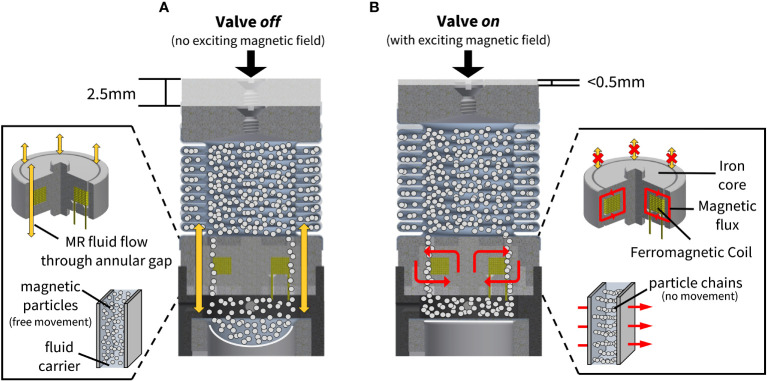
**(A)** When the valve is *off* and an external force is applied to the module, the fluid remains in its liquid state and is pressed from the deformable bellow through the flow channels and annular gap in the valve to the auxiliary reservoir below (downward yellow arrows). **(B)** When the valve is *on*, the fluid is solidified by the magnetic flux (cyclical red arrows) such that there is no fluid flow from the deformable bellow to the auxiliary reservoir below.

The design of the removable insole system is such that when pressure is applied by the foot to the area above a module (e.g., from standing or walking), the module will be triggered to operate in one of two states: 1) valve *off*, or 2) valve *on*. When the valve is *off*, the MR material remains in its fluid state and can move through the flow channels and the annular gap in the valve to be dispensed into the auxiliary reservoir (**Figure 5A**). The resultant movement of the MR material results in a maximum module compression of 2.5 mm. When the force is removed, the reflow membrane forces the fluid to return into the deformable bellow above. However, when the valve is *on*, the exciting magnetic field (magnetic flux) causes the MR material to solidify in the valve channels and prevents the fluid from traveling to the auxiliary reservoir (**Figure 5B**). In the *on* state, there is a maximum module compression of 0.5 mm due to mechanical stabilization.

**Figure 6:**
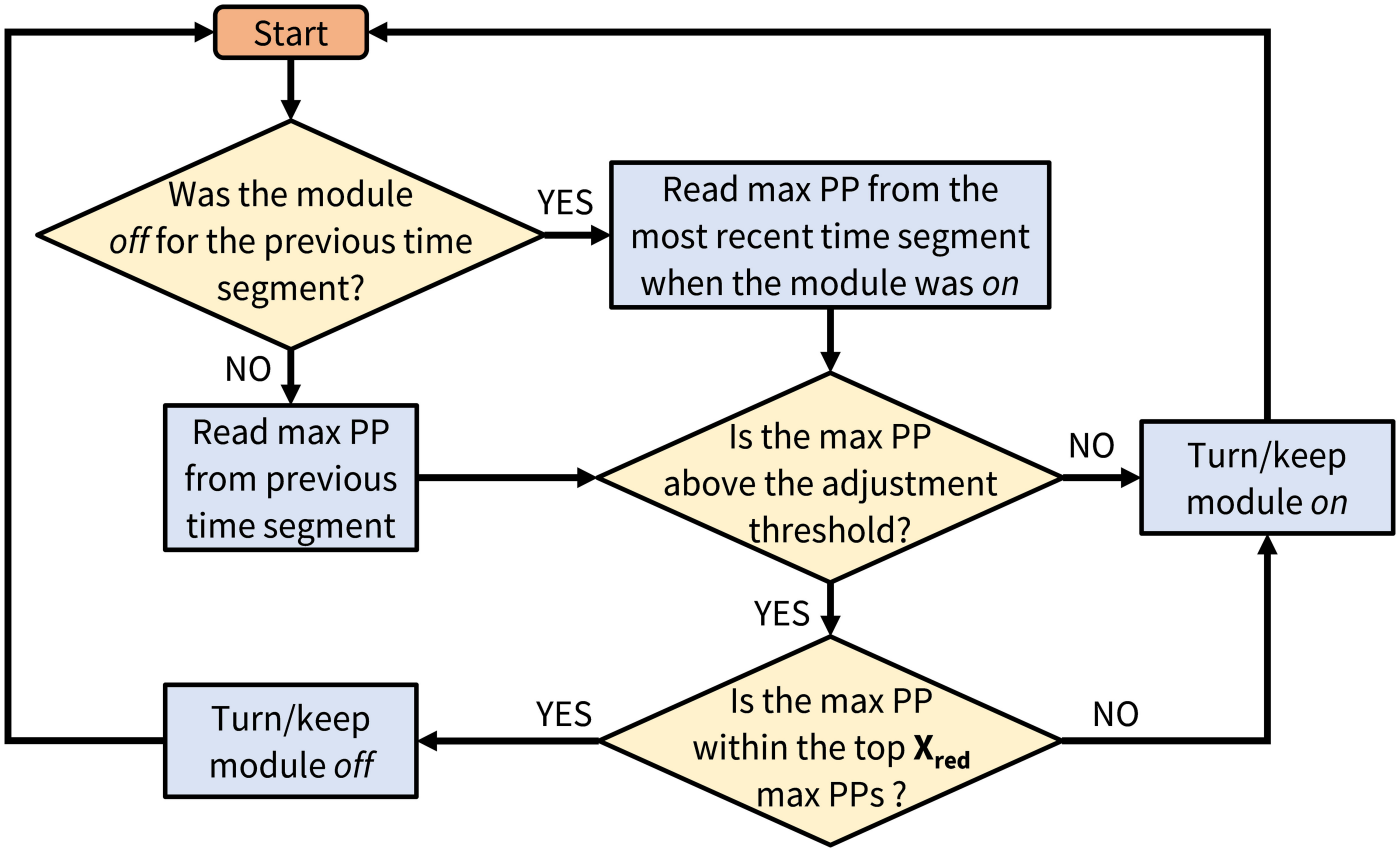
Flow diagram of the baseline decision algorithm (mPP – the maximum of the sensor’s peak PP; X_red_ – the maximum number of modules that would be turned *off* at one time).

A baseline decision algorithm (based on the maximum peak pressure sensed above a module) will be employed to determine which modules will be turned *off* according to the user’s pressure needs (**Figure 6**). There will be a set number of modules that may be turned *off* at one time (X_red_) to redistribute pressures. In the future, a trained and validated machine learning algorithm will be used to intelligently control which modules are *off* or *on*.

The design of the inner components is based on previous research and tailored to be suitable for footwear conditions. The size of the region above each module that can be deformed to achieve the intended pressure redistribution is a compromise between the complexity of the control system (in terms of both the hardware and the algorithm) and the accuracy in the determination of the magnitude and location of the peak PPs. In this respect, previous research (38) underlined that in most cases, a sensor having a surface area of 1 cm^2^ is sufficient (accuracy of *≈* 90%) to define the position and the proportions of the peaks of PPs. Thus, the reference value for the surface area exposed to loading (above each pressure-offloading module) is fixed to 1.6 cm^2^, being a compromise between sufficient sensing/actuation resolution and system complexity. Each of the modules is waterproof and future designs will incorporate this same quality for all other electrical components. Further tests will also address safety features such as componentry heating and falls risk due to the elevated height of the footwear (measurement of ground to foot plantar height = 6.3 cm).

The module’s performance and the system’s ability to reduce PP across the insole have been tested. In the module’s *on* state, it could sustain a load of 55N, which corresponds to 357 kPa (39) with a residual deformation of only 0.5 mm. Thus, the performance of the module while *on* meets performance standards; with a PP of this magnitude, the module would likely be turned *off* to offload that region to prevent a foot ulcer. When the module was turned *off* during the tests, the module instantaneously deformed to 1.5 mm which was the maximum deformation allowed for this test, and the force was reduced to 30 N (corresponding to 214 kPa) (39). This final force was linked to the features of the deformable bellow and the hydraulic resistance. Furthermore, a preliminary walking study with four, healthy, male adults was conducted to assess the PP reduction. Participants wore the prototype of the footwear with surrogate modules as they walked 10m at a comfortable walking speed. The deformation of the module between the *on* and *off* states allowed for a maximum reduction of PP of 18-24% directly over the module and 6-10% reduction in the area around the module when the peak starting PP ranged from 273-607 kPa (40). Furthermore, for cases with an initial peak PP above 400 kPa, there was a 20-32% reduction in peak PP. The present study was approved by the University Commission for Ethical Research in Geneva (CUREG 2022-07-78).

## 3 Design for patient use and adherence

Adherence is an essential aspect of medical device use. To understand the user perceptions and potential adherence barriers to this footwear, a pilot, in-person questionnaire and a larger, online questionnaire were conducted concerning the intelligent footwear presented in this article (41). Ethical approval was obtained to conduct the questionnaires (CUREG 2022-03-35). Across the two questionnaires, people with diabetes (n=48), caregivers of people with diabetes (n=10), and healthcare professionals working with people with diabetes (n=65) from 30 countries on 6 continents gave important insight regarding the functionality, potential adherence, self-image, and aesthetics of the footwear. The questionnaires addressed the potential use and barriers to using this intelligent footwear based on previous work (42, 43); questionnaires were administered and processed by the researchers with aid from clinicians. Generally, 95% of respondents thought that it would be beneficial to use the footwear and over 70% in each role stated that they would use the footwear or recommend it to their patients when available. Several parts of the questionnaire addressed self-image while wearing the footwear and perceived efficacy of using the footwear. The results informed aspects of this intelligent footwear design such as implementation of a sports shoe design which was the most preferred style among others (41). Future designs of this footwear will include styles of shoes for all occasions for men and women.

One of the limitations of other “smart” offloading footwear is the need for the patient to interact with a device and alter gait to relieve areas of high PPs (25–27, 44). To lessen the required user-involvement and thus, increase the likelihood of adherence, this intelligent footwear will have an autonomous, pressure-redistribution algorithm. As the individual wears the shoes, the insole will regularly read the PPs and automatically change the contour of the insole eliminating the need for the patient to interact directly with the device during the day. The only required interaction is the need to charge the shoes each day after wear. With a current of 0.7A, activating the *on* state of a module (200 ms) for 5,000 steps (recommended daily step count per foot for people with diabetes (45, 46)) would require 195 mAh per module. One shoe of size EU 43 has 31 modules which would require a total of ~6,000 mAh. Therefore, a battery with 9,000mAh of energy (footwear has the capacity to house two batteries) is sufficient to provide a day’s worth of charge for each shoe. To apprehend complications with charging that could possibly reduce adherence, the footwear is designed to have a charging mechanism similar to technology that users may already operate (e.g., cellphones, tablets). Furthermore, assessments of other adherence parameters have been performed and are ongoing in order to increase footwear adherence (41).

## 4 Conclusion

The presented intelligent footwear is designed to automatically and autonomously redistribute high PP under the feet of people with diabetes and specifically those with neuropathy. The mechanisms to offload the pressures use an intelligent, removable insole system which will actively adapt to the person’s foot while they are wearing the intelligent footwear. The footwear is designed to improve adherence through simplicity of user involvement and aesthetics resembling footwear not associated with a medical condition. Future versions will improve upon the technical and human factors aspects of the footwear to enhance flexibility, durability, battery life, usability, and aesthetics. The technological and adherence aspects of the footwear will continue to be tested and improved through clinical trials.

## Author contributions

YP and ZP conceived of the original idea. KJ, SN, CK and BT developed the technological aspects of the idea (modules, electronics, removable insole system, etc.). YC, SH, and ZP developed the structural design of the footwear. YC, CK, YP and ZP supervised and guided the project. SH wrote the manuscript. All authors contributed to the article and approved the submitted version.
